# Characterizing the role of an endogenous serine protease *Kp*Sub2 in recombinant collagen degradation in *Komagataella phaffii*

**DOI:** 10.1186/s40643-026-01039-y

**Published:** 2026-03-26

**Authors:** Shichang Feng, Jianfeng Zhao, Jun Chen, Feng Liu, Qiang Hua

**Affiliations:** 1 State Key Laboratory of Bioreactor Engineering, East China University of Science and Technology, 130 Meilong Road, Shanghai, 200237 China; 2 Zhejiang Zhuji JLand Biotechnology Co., Ltd., No.69 Youyi North Road, Zhejiang 311800 Zhuji, China

**Keywords:** Recombinant humanized collagen, Serine protease, Proteolytic degradation, *Komagataella phaffii*

## Abstract

The yeast *Komagataella phaffii* is an emerging microbial host for the production of functional recombinant proteins. However, proteolytic degradation during fermentation often compromises product yield and stability, posing a major hurdle for industrial-scale applications. This study presents a strategy to enhance the production of recombinant humanized type I collagen (rhColI) by engineering a host strain with reduced protease activity. An initial production strain, CL1, was engineered using post-transformational vector amplification (PTVA), achieving a titer of 558.86 ± 20.05 mg/L in flask culture. Subsequent scale-up fermentation, however, revealed significant rhColI degradation. To address this, we systematically deleted 11 candidate endogenous protease genes. The knockout of a serine protease gene, *Kp*Sub2, resulted in the most pronounced improvement, elevating the rhColI titer to 1039.06 ± 34.08 mg/L, a 23.47% increase over the parent strain CL1. Furthermore, the purified *Kp*Sub2 protein, obtained from inclusion bodies in *Escherichia coli*, demonstrated broad proteolytic activity against various types of recombinant humanized collagens. This broad substrate specificity was consistent with the observation that *Kp*Sub2 deletion also mitigated the degradation of recombinant humanized type III collagen (rhColIII). Our findings establish *Kp*Sub2 as a key mediator of collagen degradation in *K. phaffii* and provide an effective engineering strategy for optimizing the production of collagen and other degradation-susceptible functional proteins in this host.

**Graphical abstract:**

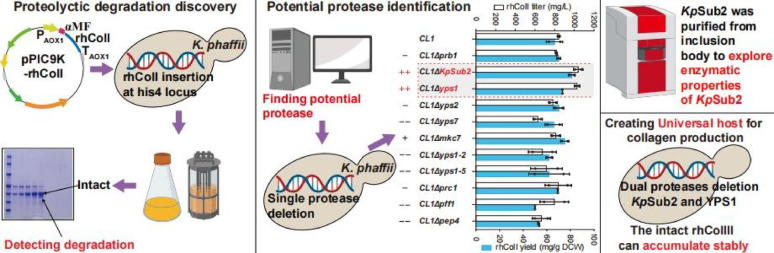

**Supplementary Information:**

The online version contains supplementary material available at 10.1186/s40643-026-01039-y.

## Introduction

Collagen, constituting approximately 25–35% of the total protein in vertebrates, serves as the primary structural scaffold in skin, bone, cartilage, blood vessels, and the cornea because of its distinctive triple-helical conformation and self-assembly properties, which confer exceptional tensile strength, resilience, and structural integrity to tissues (Fidler et al. 2018; Kurt et al. 2024). Owning to these characteristics, collagen has been extensively exploited in tissue engineering (Sorushanova et al. 2019). Beyond its mechanical function, collagen also modulates critical cellular behaviors, including adhesion, migration, proliferation, and differentiation, through interactions with cell surface receptors such as integrins (Pathak et al. 2025).

Conventional collagen production relies on the acid solubilization or enzymatic hydrolysis of animal-derived tissues (Gutierrez-Canul et al. 2025). However, this approach faces considerable limitations concerning raw material safety, process efficiency, and final yield. Synthetic biology offers a promising alternative, enabling the cost-effective and scalable production of humanized collagen (Raza et al. 2020). Among commonly used microbial hosts, *Komagataella phaffii* (formerly *Pichia pastoris*) has emerged as a robust platform for recombinant human collagen production (Myers et al. 2004; Zhao et al. 2023). Its simplified glycosylation machinery favors high-yield synthesis of recombinant human proteins and helps circumvent the issue of hyperglycosylation-induced immunogenicity often encountered with *Saccharomyces cerevisiae* (Daly and Hearn 2005). Compared to *E. coli* expression system, *K. phaffii* offers several advantages, including the absence of endotoxin and the capacity for post-translational modifications. For instance, human-like gelatin expression in GS115 reached up to 57 mg/L in shake flask culture (Song et al. 2024). Wang et al. successfully constructed the engineered *K. phaffii* strains to secrete human type II collagen variants, which demonstrated superior performance in promoting cell migration and differentiation (Wang et al. 2024). Concurrently, Yan et al. engineered *K. phaffii* to produce human type XVII collagen, achieving final titers of 2.72 g/L and 4.36 g/L in fed-batch culture, respectively (Yan et al. 2024).

Nevertheless, during high-density fermentation of *K. phaffii*, recombinant proteins are frequently susceptible to proteolytic degradation by endogenous proteases, substantially compromising both yield and product quality (Tarallo et al. 2024). Especially those with partially folded or unstructured regions, are susceptible to degradation during vacuolar transit and secretion in *K. phaffii*. The responsible proteases, belong primarily to the serine protease, aspartic protease, and vacuolar protease families (Vijayakumar and Venkataraman 2024). Specific proteases have been identified by previous studies, such as protease A Pep4, protease B Prb1, and aspartyl protease YPS1, which can degrade recombinant proteins during intracellular trafficking and secretion (Reichard et al. 2000; Salamin et al. 2010; Sazonova et al. 2013). The extent of proteolytic degradation is regulated not only by protease expression levels but also by fermentation parameters, including methanol induction time and medium composition (Shemesh and Fishman 2024). Additionally, the intrinsic structural stability and folding efficiency of the recombinant protein itself crucially influence its susceptibility to proteolysis (Raschmanová et al. 2021).

Common strategies to mitigate proteolysis include creating protease-deficient strain and optimizing fermentation conditions. However, commercially available protease-deficient *K. phaffii* strains are not universally effective and often exhibit reduced cellular productivity (Zhang et al. 2007). While controlling environmental parameters such as temperature and pH can alleviate degradation, it remains challenging to establish a single fermentation regime that simultaneously supports optimal yeast growth and maximizes recombinant protein expression (Ma et al. 2016). Moreover, high-density cultivation, particularly during methanol induction, can induce cell lysis, releasing intracellular proteases into the culture supernatant and thereby accelerating degradation (Scorer et al. 1993). Therefore, developing a tailored chassis strain optimized for specific protein classes represents a compelling alternative.

In this study, we aim to engineer *K. phaffii* for high-yield and stable production of recombinant humanized collagen. Using post-transformational vector amplification (PTVA), we first constructed strains carrying high-copy expression cassettes for recombinant humanized type I collagen (rhColI) and type III collagen (rhColIII). Although high-level expression was achieved, significant degradation was observed during scale-up fermentation. To address this, we systematically identified and individually deleted 11 candidate endogenous protease genes. Among these, the serine protease *Kp*Sub2 (a homolog to Prb1) and the aspartic protease YPS1 were found to be primarily responsible for rhColI degradation. We further characterized the enzymatic properties of *Kp*Sub2, including its activity towards different types of collagen and its response to temperature, pH, and metal ions. Moreover, we demonstrated that deletion of *Kp*Sub2 enhances the production stability of rhColIII. This work establishes a robust foundation for enhancing recombinant collagen production in *K. phaffii* expression system.

## Results and discussion

### Efficient expression of rhColI in ***K. phaffii***

**Fig. 1:**
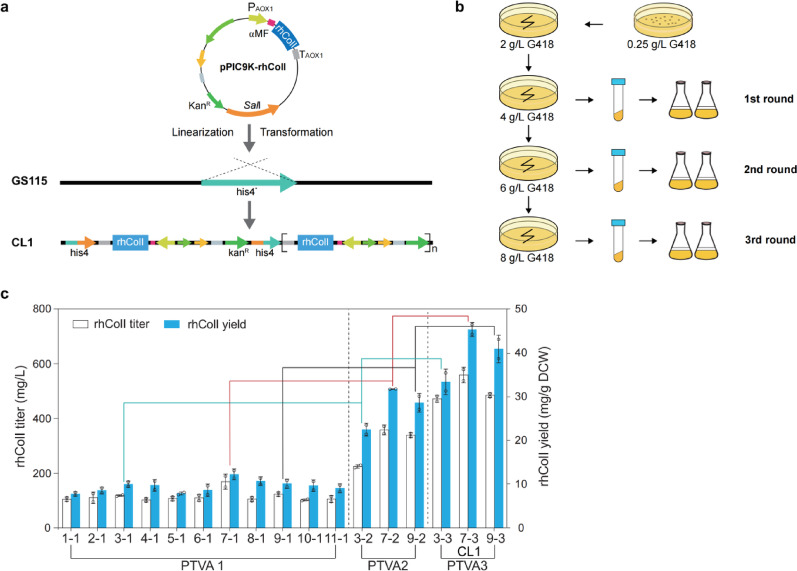
Plasmid construction and strain selection for the engineered rhColI-producing strain. **a** Schematic of the expression vector construction, linearization and transformation for rhColI. **b** Workflow for selecting multicopy clones enriched by post transformational vector amplification (PTVA) at G418 concentrations ranging from 0.25 to 8 mg/mL incubated at 30 °C. **c** rhColI production titer and yield of engineered strains after 3 days methanol induction during the PTVA process

The rhColI sequence was designed based on functional domains of the human collagen alpha-1(I) chain. The strategies for plasmid construction and multicopy clone screening are illustrated in Fig. 1a and b, respectively. Here we employed the PTVA method to generate and select *K. phaffii* clones harboring multiple copies of the expression cassette (Jiao et al. 2018). Increasing the G418 concentration at each PTVA round enriched clones with higher rhColI copy numbers. To validate the efficacy of this approach, the production profiles of selected *K. phaffii* clones were analyzed by HPLC after 3 days of methanol induction (Fig. 1c). The maximum rhColI titers achieved in three successive screening rounds were 169.53 mg/L (strain 7-1), 358.57 mg/L (strain 7-2), and 558.86 mg/L (strain 7-3). The strain CL1, integrated with 8 copies of the rhColI cassette at *his4* locus, was selected as the high-yield producer for subsequent studies. These results confirm the effectiveness of PTVA for screening clones with elevated gene copy numbers.

In flask cultures, a primary degradation band was observed, with minimal subsequent degradation, particularly in high-producing strains. However, substantial further degradation was observed in a 100 L bioreactor. HPLC analysis revealed that the area percentage of the principal rhColI fragment decreased from 47% at 72 h to 6% by 112 h of fermentation (Fig. S1). We propose that proteases expressed at low level play a critical role in rhColI degradation, as their progressive accumulation during high-density fermentation exacerbates collagen hydrolysis.

### Identification of the endogenous ***Kp***Sub2 involved in rhColI degradation

**Fig. 2:**
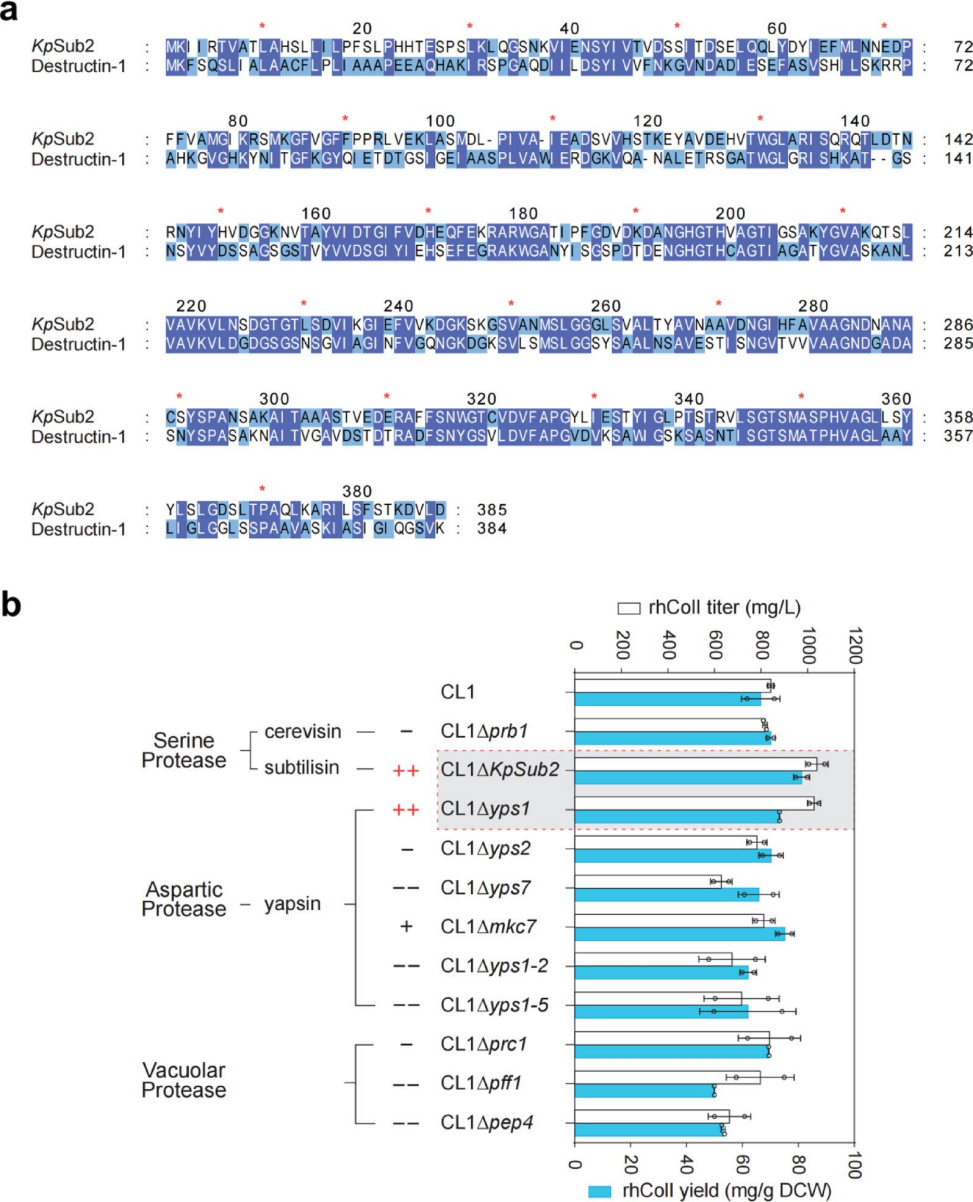
Identification of protease deletion targets to mitigate rhColI degradation. **a** Amino acid sequence alignment between *Kp*Sub2 and the reported collagenase Destructin-1 from *Pseudogymnoascus destructans*. **b** rhColI production in shake flask cultures of various proteinase-deficient strains derived from the parent strain CL1

To facilitate downstream purification by minimizing rhColI degradation in pilot-scale cultures, we selected eleven candidate proteases from serine, aspartic, and vacuolar protease families to be individually knocked out in strain CL1. Among these, the subtilisin-like protease *Kp*Sub2 was prioritized by its sequence alignment with Destructin-1, a microbial collagenase from *Pseudogymnoascus destructans* (O’Donoghue et al. 2015). As shown in Fig. 2a, *Kp*Sub2 shares 40.55% sequence identity with Destructin-1. To simulate degradation conditions akin to pilot-scale cultures, shake flask cultures were extended to 5 days to promote rhColI proteolysis. Under these extended shake flask cultivation conditions, the rhColI titer of strain CL1 reached 847.57 ± 9.00 mg/L. Deletion of *Kp*Sub2 enhanced the rhColI titer in shake flasks to 1039.06 ± 34.08 mg/L, representing a 23.47% increase over the parental strain CL1 (Fig. 2b). Previous studies have demonstrated that subtilisin family proteases exhibit potent collagen-degrading activity by cleaving glycine-containing peptide bonds, which are abundant in collagen (Ran et al. 2013; Cheng et al. 2023). Our results further validated that *Kp*Sub2 possesses the collagenase activity in *K. phaffi*. Moreover, *YPS1* deletion also improved the titer to 1027.94 ± 20.05 mg/L. Different from *Kp*Sub2, the disruption of *YPS1* serves as a routine strategy to mitigate the proteolytic degradation of secreted heterologous proteins, including collagen (Werten and de Wolf 2005; Wang et al. 2023), human serum albumin (HSA) and parathyroid hormone (PTH) (Wu et al. 2013), consequently enhancing their stability and overall production yield.

To assess the impact of protease deletion on cell growth, we cultivated CL1, *Kp*Sub2-deficient, and YPS1-deficient strains in buffered complex medium with glucose, glycerol, and methanol as the sole carbon source (Fig. S2). The biomass of these mutant strains showed no significant difference. In contrast, deletions of some proteases reduced rhColI production, potentially due to adverse effects on cell growth. The observed production decrease may be linked to the reduced efficiency of the corresponding protease in utilizing complex nitrogen sources, such as tryptone. This disturbed nutrient uptake led to lower biomass (Fig. S3), and reduced metabolic fitness in complex fermentation medium, ultimately impairing product synthesis. In *S. cerevisiae*, *Pep4* deletion resulted in the decreased accumulation of pyruvate, an key metabolic intermediate, further affecting overall metabolism in yeast (Carmona-Gutiérrez et al. 2011; Hu et al. 2019). Even though the *Pep4* disruption almost had no effect on cell biomass as strains were cultivated in BMMY medium, the *YPS7*- and *PRC1*-deficient strains exhibited significantly reduced biomass accumulation compared to other strains (Fig. S3). According to the previous study, YPS7 plays a pivotal role in degrading deleterious proteins and maintaining vacuolar homeostasis, particularly under stress conditions (Vidal-Montiel et al. 2025). The deficiency of vacuolar proteases, including PRC1, may impair the efficient recycling of amino acids via autophagy, thereby significantly compromising cellular viability and physiological fitness.

### Enzymatic characterization of ***Kp***Sub2 toward collagen

**Fig. 3:**
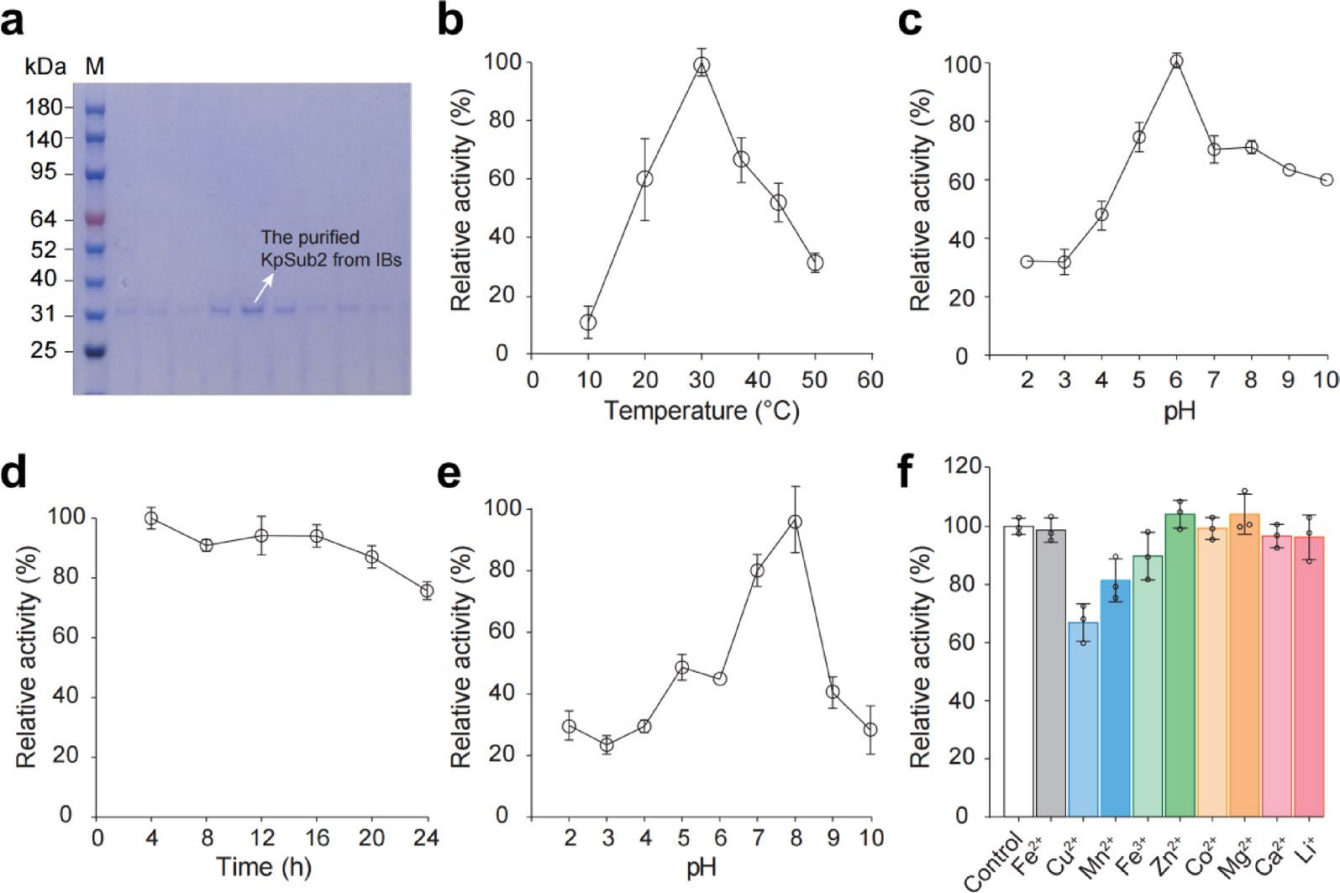
Enzymatic characterization of *Kp*Sub2. **a** SDS-PAGE analysis of *Kp*Sub2 purified by nickel affinity chromatography. M: prestained protein molecular weight marker. **b** Effect of temperature on the proteolytic activity of *Kp*Sub2 toward rhColI. Assay were conducted in 20 mM PBS (pH 8.0). The highest enzyme activity (observed at 30 °C) was defined as 100%, and the relative enzyme activities at other temperatures were calculated accordingly. **c** Effect of pH on proteolytic activity of *Kp*Sub2 toward rhColI. The highest enzyme activity (observed at pH 6.0) was defined as 100%, and the relative enzyme activities at other pH values were calculated accordingly. **d** Thermal stability of *Kp*Sub2. Residual activity was measured after incubation in 20 mM PBS (pH 8.0) at 30 °C for 24 h, with the initial activity (at 0 h) defined as 100%. **e** pH stability of *Kp*Sub2. Residual activity was measured after incubation at different pH values at 4 °C overnight. Activity after incubation at pH 8.0 was defined as 100%. **f** Effects of metal ions (5 mM) on *Kp*Sub2 activity. Activity in the absence of metal ions was determined as 100%

Although *Kp*Sub2 was implicated in rhColI proteolysis, its enzymatic properties, particularly toward collagen substrates, remained poorly characterized. *Kp*Sub2 is known to be secreted via a signal peptide. Previous attempts to express *Kp*Sub2 in *K. phaffii* GS115 yielded only 3 ng/mL in the cell supernatant (Salamin et al. 2010). In this study, the engineered *K. phaffii* strain also produced insufficient *Kp*Sub2. Therefore, we utilized *E. coli* BL21(DE3) as an alternative host for the heterologous production of both truncated and full-length *Kp*Sub2. Both constructs predominantly accumulated in form of inclusion bodies (IBs), even as fused to solubility-enhancing tags such as small ubiquitin-related modifier (SUMO) or maltose-binding protein (MBP). Nevertheless, we successfully purified *Kp*Sub2 from IBs. SDS-PAGE analysis confirmed the presence of a protein band near the 34 kDa marker (Fig. 3a).

To investigate the substrate specificity of *Kp*Sub2, we evaluated its proteolytic activity against several types of recombinant humanized collagens. As summarized in Table S1, *Kp*Sub2 exhibited detectable, though relatively low, activity against rhColI, rhColIV, and rhColVII, with the highest activity observed toward rhColI, which may be attributed to the high frequency of A-G peptide bonds (up to 30) in rhColI. From a structural perspective, the catalytic mechanism of subtilisin-like serine proteases is often dictated by the geometry of their active site pockets. Their pockets tend to preferentially accommodate amino acids with small steric hindrance, such as glycine and alanine, allowing them to fit into the catalytic cleft for cleavage (Cheng et al. 2023). This structural constraint explains why *Kp*Sub2 exhibits potent hydrolytic activity against glycine-rich proteins like collagen. Interestingly, the other tested types of collagen lack A-G peptide bonds (as shown in Table S5). We speculated that the enzymatic activity of *Kp*Sub2 against these substrates results from hydrolysis of G-P peptide bonds, consistent with previous reports that collagenase can also cleave G-P bonds (Ran et al. 2014; Cheng et al. 2023).

Subsequently, rhColI was selected as the model substrate for further characterization. As shown in Fig. 3b, *Kp*Sub2 retained over 80% of its enzymatic activity within the temperature range of 20–37 °C, with maximum relative activity observed at 30 °C, which is near the standard cultivation temperature of 28 °C used for *K. phaffii*. The optimum pH for activity was determined to be pH 6 (Fig. 3c). Moreover, the enzyme maintained more than 60% of its activity after 24 h of incubation at 30 °C (Fig. 3d), and exhibited sustained activity across a broad pH range of pH 6–8 (Fig. 3e). Regarding metal ion effects, only Cu^2+^, Mn^2+^, and Fe^3+^ significantly inhibited enzymatic activity, with Cu^2+^ showing the strongest suppression (Fig. 3f). The significant inhibition of *Kp*Sub2 by Cu^2+^, Mn^2+^, and Fe^3+^ is consistent with the known behavior of transition metals as protease inhibitors. These cations likely coordinate with key active site residues, such as the histidine and aspartate of the catalytic triad (Patel et al. 2023). This binding, for which Cu^2+^ often shows high affinity, can disrupt the precise catalytic geometry or sterically block substrate access, thereby abrogating enzymatic function (Majeed et al. 2024).

Collectively, these findings demonstrate that *Kp*Sub2 maintains relatively high proteolytic activity against rhColI under conditions mimicking the *K. phaffii* fermentation environment, supporting its potential role in collagen degradation in this host. While the *E. coli*-derived enzyme lacks the complex post-translational modifications inherent to eukaryotic systems, we posit that these modifications primarily modulate the absolute activity levels or environmental stability rather than fundamentally altering the enzyme’s core catalytic mechanism. The preference for cleaving glycine-adjacent peptide bonds is fundamentally determined by the structural geometry of the *Kp*Sub2 catalytic pocket. Thus, the enzymatic characterization of the prokaryotic recombinant *Kp*Sub2 effectively captures the essential biochemical properties relevant to collagen proteolysis in yeast hosts.

### Inactivation of ***Kp***Sub2 enhances the stability of rhColIII

**Fig. 4:**
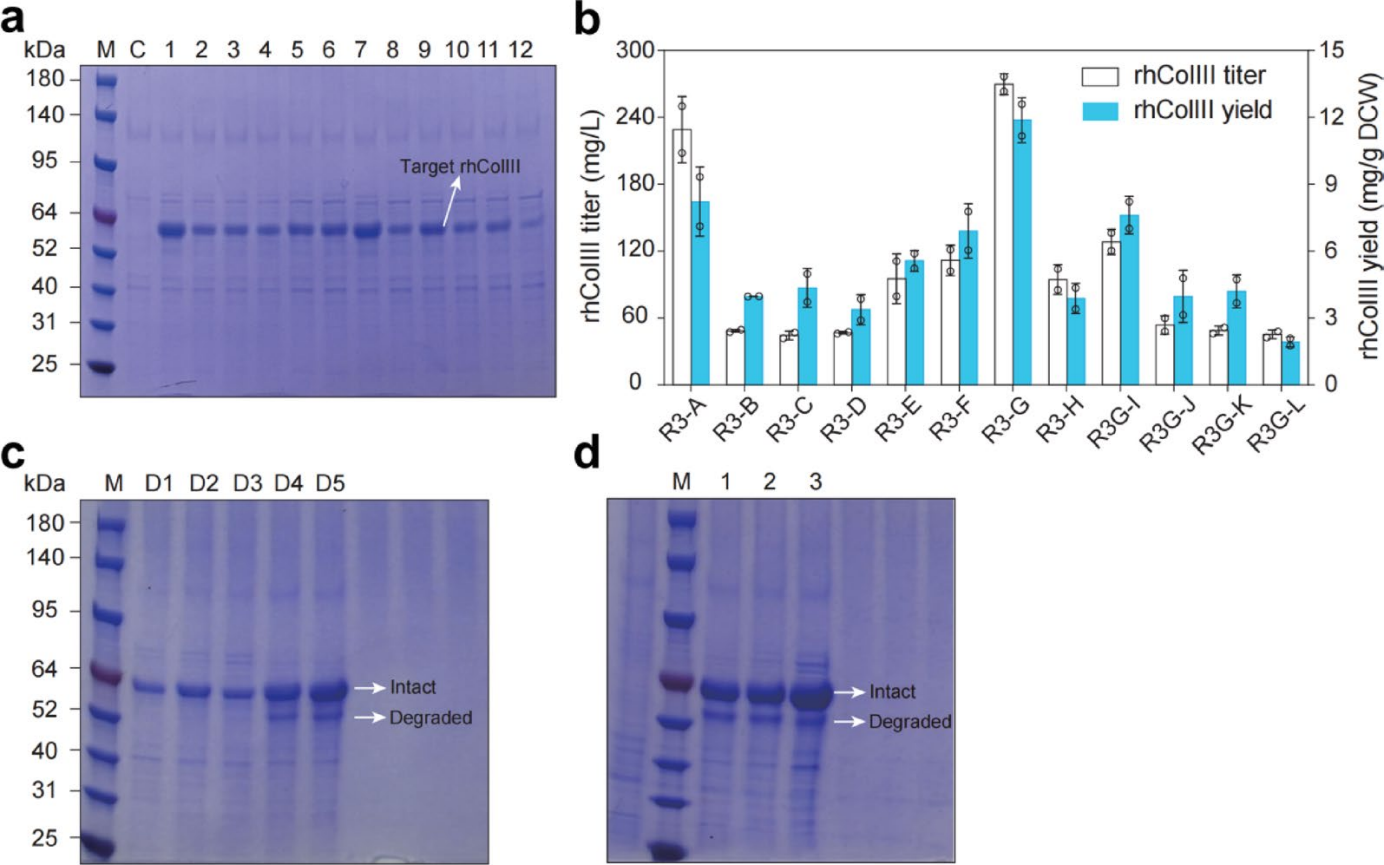
Production performance of rhColIII in engineered strains. **a** SDS-PAGE analysis for strains screened for high rhCOlIII production via PTVA method for 3 days of fermentation. M: prestained protein molecular weight marker; C: GS115 transformed with linearized empty pPIC9K vector; lanes 1–12, engineered strains R3-A to R3-L. **b** rhColIII production titers of different PTVA-screened strains after 3 days of fermentation. **c** Time-course analysis of secreted rhColIII degradation from day 1 to day 5 of fermentation. **d** SDS-PAGE analysis of rhColIII production in protease-deficient strains after 4 days of fermentation. M: prestained protein molecular weight marker; C: GS115 transformed with linearized empty pPIC9K vector; Lane1: strain R3-G, Lane2: strain R3-G1 (*Yps1* knockout), Lane3: strain R3-G2 (*Yps1* and *Kp*Sub2 double knockout)

Similarly, a functional fragment derived from the human collagen alpha-1(III) chain was designed, codon-optimized, and expressed in *K. phaffii*. Using the PTVA method, the engineered strain R3-G was isolated and shown to produce 269.98 ± 6.71 mg/L of recombinant human collagen type III (rhColIII) (Fig. 4a and b). qPCR analysis confirmed the integration of three copies of the rhColIII gene at the *his4* locus. Analogous to the behavior observed with rhColI, degradation of rhColIII initiated during the late stages of fermentation and progressed until the process endpoint (Fig. 4c). To enhance rhColIII production, we applied a targeted protease deletion strategy in strain R3-G, focusing on the previously identified proteases *Kp*Sub2 and YPS1. While integration of the rhColIII expression cassette at the *yps1* locus was successful (yielding strain R3-G1), integration at the *Kp*Sub2 locus was not achieved. To evaluate potential synergistic effects, *Kp*Sub2 was subsequently disrupted in R3-G1, resulting in the double-knockout strain R3-G2. As shown in Fig. 4d, although the copy number of rhColIII was increased in R3-G1, significant degradation persisted. In contrast, the additional deletion of *Kp*Sub2 in R3-G2 nearly eliminated rhColIII degradation, thereby markedly promoting its stable accumulation. These results indicate that *Kp*Sub2 likely mediates collagen degradation through specific recognition of collagen-like sequences.

## Conclusions

In summary, we have, for the first time, screened and identified an exogenous serine protease, *Kp*Sub2, that significantly contributes to the proteolysis of recombinant collagen (rhColI) during expression in *K. phaffii*. By purifying from inclusion bodies in *E. coli*, we confirmed its ability to degrade various types of recombinant collagen. Furthermore inactivation of *Kp*Sub2 enhanced recombinant collagen stability, promoting accumulation of intact product. Mechanistically, the catalytic pocket of *Kp*Sub2 is predicted to preferentially accommodate amino acids with small steric hindrance, such as glycine. Therefore, we propose that this *Kp*Sub2-deficient strain is particularly well-suited as a specialized host for the production of other glycine-rich structural proteins, such as silk fibroin and elastin, which share similar sequence characteristics with collagen.

## Materials and methods

### Chemicals, reagents, and strains

All the humanized collagen substrates were provided by Zhejiang Zhuji JLand Biotechnology Co., Ltd. Tryptone and yeast extract were purchased from Oxoid (Hampshire, UK). Urea was purchased from Shanghai Haohong Biomedical Technology Co., Ltd. Yeast nitrogen base without amino acids, antibiotics (Amplicilin, kanamycin, geneticin and zeocin), and Isopropyl β-D-1-thiogalactopyranoside (IPTG) were purchased by Sangon Biotech (Shanghai) Co., Ltd. Gelatin, other buffers and solvents were obtained from Shanghai Macklin Biochemical Co., Ltd.

All the primers used in this study were synthesized by Beijing Tsingke Biotechnology Co., Ltd. The genes for rhColI and rhColIII were designed based on specific functional domains derived from the human collagen alpha-1(I) and alpha-1(III) chains, respectively. These genes do not represent full-length sequences but are spliced functional fragments optimized for heterologous expression in *K. phaffii*. The amino sequences of rhColI, rhColIV, rhColVII were listed on Table S5.

The optimized genes were synthesized by Beijing Tsingke Biotechnology Co., Ltd. Gene fragments were amplified using TransStart^®^ FastPfu Fly DNA Polymerase (TransGen Biotech, Beijing, China). Colony PCR screening was carried out using 2× Rapid Taq Master Mix (Vazyme Biotech, Nanjing, China). Plasmid assembly was performed via Gibson assembly using the ClonExpress Ultra One Step Cloning Kit V2 (Vazyme Biotech). All constructed plasmids were confirmed by Sanger sequencing (Jie Li Biology, Shanghai, China).

*Escherichia coli* JM109 was used for plasmid propagation, and *E. coli* BL21 (DE3) was used for heterologous protein expression. *Komagataella phaffii* GS115 was used as the host for recombinant collagen production. Strains used in this study are listed in Table S2.

### Culture conditions

*Escherichia coli* strains were cultivated at 37 °C in Luria-Bertani (LB) medium (1% tryptone, 0.5% yeast extract, 0.5% NaCl) supplemented with 100 µg/mL ampicillin or 50 µg/mL kanamycin as required. For protein expression in *E. coli* BL21 (DE3), cells were grown to an OD_600_ of 0.5–0.8, induced with 0.2 mM IPTG, and incubated overnight at 16 °C.

*Komagataella phaffii* strains were cultured at 30 °C in YPD (1% yeast extract, 2% peptone, 2% glucose) or Minimal Glycerol (MGY; 1.34% YNB, 1% glycerol) medium.

### Plasmids construction

All plasmids and primers used for plasmid construction, including the specific vector backbones and sequences for collagen expression and gene deletion, are listed in Tables S3 and S4, respectively.

### Expression plasmids

The vectors pPIC9K (*K. phaffii*) and pET28a (*E. coli*) served as backbones for protein expression. The synthesized *rhColI* and *rhColIII* gene fragments were cloned into pPIC9K by Gibson assembly, respectively. The obtained *Kp*Sub2 gene fragment was amplified and cloned into the expression plasimd pET28a for expression in *E. coli*.

### Plasmids construction for single gene deletion and Insertion

The CRIPSR/Cas9 system used in this study was established by Prof. Menghao Cai from East China University of Science and Technology (Liu et al. 2019). The pPIC3.5 K vector, containing Zeocin antibiotic expression cassette, was used as the backbone plasmid for Cas9 expression and guide RNA (gRNA) delivery. All gRNAs were designed using the CHOPCHOP webtool (http://chopchop.cbu.uib.no). An *K. phaffii* exogenous artificial replication sequence enabled the stable CRIPSR/Cas9 system activation.

Donor DNA plasmids were constructed based on the pUC19 vector. For gene deletions, 1000 bp upstream and downstream homologous arms flanking the target sequence were fused into pUC19. For gene insertion, the upstream homologous arm, the target expression cassette, and the downstream homologous arm were fused into pUC19 via Overlap Extension PCR and Gibson assembly.

### ***K. phaffii*** electroporation and transformant selection

For transformation of collagen genes, approximately 1 µg of linearized plasmid DNA (pPIC9K-*rhColI* or pPIC9K-*rhColIII* linearized with *Sal*I) was mixed with an 100 µL aliquot of the competent cell suspension. As for single gene deletion or insertion, 100 ng gRNA plasmids and 1000 ng donor DNA fragments were enough for efficient transformation.

The DNA-cell mixture was transferred to an ice-cold 0.2 cm gap electroporation cuvette and incubated on ice for 5 min. An electrical pulse was delivered using an electroporator (Bio-Rad Gene Pulser Xcell) with the following parameters: 1.5 kV, 25 µF, and 200 Ω. Immediately following the pulse, 0.5 mL of ice-cold 1 M sorbitol was added to the cuvette to recover the cells. The cell suspension was transferred to a sterile centrifuge tube with 0.5 mL of fresh YPD medium. After 2–4 h recovery in 30 °C (220 rpm), 100–200 µL aliquots of the cell suspension were plated onto selective media (YPD supplemented with 100 µg/mL Zeocin or MGY supplemented with 0.25 mg/mL G418 plates). Plates were incubated at 30 °C for 3–5 days until colonies were visible. Following validation by yeast colony PCR, the sgRNA plasmid was cured by cultivating positive clones on non-selective YPD plates.

### ***K. phaffii*strain engineering and screening**

Multicopy clones of *K. phaffii* were generated and screened using the post-transformational vector amplification (PTVA), as previously reported (Jiao et al. 2018). Initially, transformants were selected on MGY agar plates containing 0.25 mg/mL G418. Positive clones were then subjected to successive rounds of enrichment on YPD agar plates with an increasing G418 concentration gradient of 2.0, 4.0, 6.0, and 8.0 mg/mL. For each round, the plates were incubated at 30 for 3–5 days until well-defined colonies were visible. Clones exhibiting the highest resistance and production levels of rhColI and rhColIII were then isolated for further evaluation.

### Shake flask fermentation

For recombinant protein expression, a single colony of the engineered *K. phaffii* strain was inoculated into 10 mL of YPD medium and cultured overnight at 30 °C with agitation (220 rpm).

This seed culture was used to inoculate 50 mL of BMGY medium (Buffered Glycerol-Complex Medium: 1% yeast extract, 2% tryptone, 100 mM potassium phosphate buffer pH 6.0, 1.34% YNB, 4 × 10^− 5^% biotin, and 1% glycerol) in a 250 mL baffled flask to an initial optical density (OD_600_) of 0.1. The culture was grown at 30 °C with 220 rpm shaking for approximately 18–24 h to accumulate biomass, typically reaching an OD_600_ of 2.0–6.0.

To induce protein expression, the cells were harvested by centrifugation (5000 × g, 5 min, 4 °C). The supernatant was decanted, and the cell pellet was resuspended in BMMY medium (Buffered Methanol-Complex Medium; identical to BMGY, but replacing 1% glycerol with 0.5% (v/v) methanol) to the original culture volume.

The flasks were returned to the 30 °C incubator (220 rpm). Methanol induction was initiated at a concentration of 0.5% (v/v) in BMMY to minimize toxic effects on host cells. For maintaining the induction pressure, sterile methanol was then added to the culture every 24 h to a final concentration of 1.0% (v/v). Culture samples were collected at 24-h intervals (e.g., 24, 48, 72, 96 h) for analysis. For secreted proteins, the supernatant was separated from the cell pellet by centrifugation (12,000 × g, 10 min, 4 °C) and stored at − 20 °C for subsequent analysis.

### Scale-up fermentation

Large-scale production was performed using a 100 L bioreactor (T&J Bioengineering, Shanghai, China). The engineered strain was initially grown in BMGY medium for 8 h, followed by inoculation at a 5% (v/v) ratio into a Basal Salt Medium (BSM). The BSM formulation consisted of 2.67% H_3_PO_4_, 0.094% CaSO_4_·2H_2_O, 1.82% K_2_SO_4_, 1.49% MgSO_4_·7H_2_O, 0.413% KOH, 4% glycerol, and 0.435% PTM1. Cultivation parameters were initially set at an agitation speed of 500 rpm, an aeration rate of 1 vvm, and pH 6.0.

Upon the dissolved oxygen (DO) rising to 80%, agitation was increased to 800 rpm, and a glycerol feed (50% w/w glycerol supplemented with 1.2% v/v PTM1) was introduced at 15 mL/h/L. Feeding continued until a wet cell weight (WCW) of approximately 200 g/L was achieved. Following glycerol exhaustion, the culture underwent a 1 h starvation period to ensure complete carbon source depletion before initiating induction. Methanol induction was managed using a DO-stat feeding strategy, maintaining the DO level above 20% throughout the process. The fermentation was concluded after 120 h, and the target protein expression was evaluated via HPLC and SDS-PAGE analysis.

### Heterologous expression and purification of *Kp*Sub2

Recombinant *Kp*Sub2 was expressed in *E. coli* BL21(DE3) and accumulated predominantly in inclusion bodies (IBs). These IBs were harvested and solubilized overnight at 4 °C in phosphate-buffered saline (PBS; 20 mM, pH 8.0) containing 8 M urea. The solubilized protein was then purified under denaturing conditions using Ni^2+^-affinity chromatography. Purified *Kp*Sub2 was refolded by stepwise dialysis against PBS with a gradual reduction of urea concentration from 8 to 0 M. Finally, the refolded protein solution was concentrated using an ultrafiltration device with a 10 kDa molecular weight cut-off (MWCO).

## Analytical methods

### Gene copy number determination

The copy numbers of the integrated *rhColI* and *rhColIII* genes were determined by real-time quantitative PCR (qPCR) method (Wang et al. 2025). Genomic DNA was extracted using the Yeast Genomic DNA Extraction Kit (Generay Biotech, Shanghai, China). The *rhColI* and *rhColIII* genes were amplified as target sequences, while the endogenous *GAPDH* gene was chosen as the reference. qPCR was performed on a CFX96 Real-Time system (Bio-Rad, Richmond, CA) with Hieff Unicon^®^ qPCR SYBR Green Master Mix (Yeasen, Shanghai, China). All primers used in this assay are listed in Table S4.

### Enzymatic characterization of *Kp*Sub2

The proteolytic activity of *Kp*Sub2 against recombinant humanized collagen was quantified using a ninhydrin-based assay, which detects released glycine residues (Cheng et al. 2021). The standard reaction mixture, comprising 1 mL of *Kp*Sub2 solution and 5 mg of recombinant collagen substrate, was incubated at 37 °C for 1 h with continuous stirring. One unit (U) of enzyme activity was defined as the amount of enzyme required to release 1 µmol glycine per hour from the collagen substrates. The substrates included recombinant humanized type I, IV, and VII collagens.

To determine the optimal reaction temperature, assays were conducted in 20 mM PBS (pH 8.0) across a temperature range of 10 ~ 50 °C (10, 20, 30, 40, and 50 °C) for 1 h. The pH dependence of *Kp*Sub2 was assessed at its optimal temperature using the following buffers systems: 20 mM sodium acetate (pH 2.0–5.0), 20 mM PBS (pH 6.0–8.0), and 20 mM glycine-NaOH (pH 9.0–10.0). To evaluate the effects of metal ions, *Kp*Sub2 was pre-incubated for 1 h at the optimal pH and temperature with 5 mM of various metal ions (Fe^2+^, Fe^3+^, Cu^2+^, Mn^2+^, Zn^2+^, Co^2+^, Mg^2+^, Ca^2+^, Li^+^) prior to the activity measurement.

### Quantification of rhColI

The concentration of rhColI in culture supernatants was determined by high-performance liquid chromatography (HPLC). Samples were prepared by centrifuging cultures at 12,000 × g for 10 min, and the resulting supernatant was filtered through a 0.22 μm membrane. Quantification was performed using a Thermo Scientific SEC-300 column (5 μm, 7.8 × 300 mm) with UV detection at 220 nm. The mobile phase consisted of 0.1 M phosphate buffer containing 10% (v/v) acetonitrile, delivered at a constant flow rate of 0.5 mL/min.

## Supplementary Information

Below is the link to the electronic supplementary material.

Supplementary Material 1

## Data Availability

All data generated or analyzed during this study are included in this article.

## Abbreviations

PTVA: Post-transformational vector amplification
rhColI: Recombinant humanized type I collagen
rhColIII: Recombinant humanized type III collagen
sgRNA: Synthetic guide RNA
qPCR: Quantitative PCR
IBs: Inclusion bodies
HPLC: High-performance liquid chromatography
MWCO: Molecular weight cut-off
