# Supplementary material for: Characterizing the role of an endogenous serine protease KpSub2 in recombinant collagen degradation in Komagataella phaffii
Source: Bioresour Bioprocess. 2026 Mar 26;13(1):40. doi: 10.1186/s40643-026-01039-y (PMC13022228; doi:10.1186/s40643-026-01039-y)
Supplement: Supplementary file 1 — Supplementary Material 1 [file 40643_2026_1039_MOESM1_ESM.docx]

# ****Supporting Information for****

# ****Characterizing the role of an endogenous serine protease *Kp*Sub2 in recombinant collagen degradation in****Komagataella phaffii

Shichang Feng ^1^, Jianfeng Zhao ^2^, Jun Chen ^1^, Feng Liu ^1*^, Qiang Hua ^1*^

^1^ State Key Laboratory of Bioreactor Engineering, East China University of Science and Technology, 130 Meilong Road, Shanghai 200237, China

^2^ Zhejiang Zhuji JLand Biotechnology Co. Ltd., No.69 Youyi North Road, Zhejiang 311800, China

***Corresponding author**

**E-mail address:** [qhua@ecust.edu.cn](mailto:qhua@ecust.edu.cn) (Q. Hua).

**E-mail address:** fengliu@ecust.edu.cn (F. Liu).

**Table S1.** Proteolytic activity of *Kp*Sub2 against different collagens

| Substrate | Specific activity (U/mg) |
| --- | --- |
| rhColI | 191.16 ± 20.53 |
| rhColIV | 125.30 ± 45.39 |
| rhColVII | 87.01 ± 56.23 |
| Gelatin | 115.66 ±49.01 |

**Table S2.** Yeast strains used in this study

| **Strain** | **Description** | **Source** |
| --- | --- | --- |
| GS115 | Mut+, his4-, AOX1, AOX2 | Prof. Menghao Cai |
| CL1 | GS115, *his4::*P*_AOX1_*-*rhColI*-T*_AOX1_* | This study |
| CL1-Δ*prb1* | CL1, Δ*prb1* | This study |
| CL1-Δ*KpSub2* | CL1, Δ*KpSub2* | This study |
| CL1-Δ*yps1* | CL1, Δ*yps1* | This study |
| CL1-Δ*yps2* | CL1, Δ*yps2* | This study |
| CL1-Δ*yps7* | CL1, Δ*yps7* | This study |
| CL1-Δ*mkc7* | CL1, Δ*mkc7* | This study |
| CL1-Δ*yps1-2* | CL1, Δ*yps1-2* | This study |
| CL1-Δ*yps1-5* | CL1, Δ*yps1-5* | This study |
| CL1-Δ*prc1* | CL1, Δ*prc1* | This study |
| CL1-Δ*pff1* | CL1, Δ*pff1* | This study |
| CL1-Δ*pep4* | CL1, Δ*pep4* | This study |
| R3-G | *his4::*P*_AOX1_*-*rhColIII*-T*_AOX1_* | This study |
| R3-G1 | R3-G, *yps1::*P*_AOX1_*-*rhColIII*-T*_AOX1_* | This study |
| R3-G2 | R3-G1, Δ*KpSub2* | This study |

**Table S3.** Plasmids used in this study

| **Plasmid** | **Description** | **Source** |
| --- | --- | --- |
| pPIC9K-*rhColI* | pPIC9K, AmpR, KanR, *His4*, P*_AOX1_-rhColI-*T*_AOX1_* | This study |
| pPIC9K-*rhColIII* | pPIC9K, AmpR, KanR, *His4*, P*_AOX1_-rhColIII-*T*_AOX1_* | This study |
| pUC19-*GAPDH* | pUC19, AmpR, *GAPDH* | This study |
| pUC19-*rhColI* | pUC19, AmpR, *rhColI* | This study |
| pUC19-*rhColIII* | pUC19, AmpR, *rhColIII* | This study |
| pPIC3.5K-gRNA | pPIC3.5K, Zeocin, AmpR, *His4*, P*_HTX1_*-Cas9/HH-N20-HDV | Prof. Menghao Cai |
| pPIC3.5K-Prb1-gRNA | pPIC3.5K, Zeocin, AmpR, *His4*, P*_HTX1_*-Cas9/HH-Prb1 (N20)-HDV | This study |
| pPIC3.5K-*Kp*Sub2-gRNA | pPIC3.5K, Zeocin, AmpR, *His4*, P*_HTX1_*-Cas9/HH-*Kp*Sub2 (N20)-HDV | This study |
| pPIC3.5K-Yps1-gRNA | pPIC3.5K, Zeocin, AmpR, *His4*, P*_HTX1_*-Cas9/HH-Yps1 (N20)-HDV | This study |
| pPIC3.5K-Yps2-gRNA | pPIC3.5K, Zeocin, AmpR, *His4*, P*_HTX1_*-Cas9/HH-Yps2 (N20)-HDV | This study |
| pPIC3.5K-Yps7-gRNA | pPIC3.5K, Zeocin, AmpR, *His4*, P*_HTX1_*-Cas9/HH-Yps7 (N20)-HDV | This study |
| pPIC3.5K-Yps7-gRNA | pPic3.5K, Zeocin, AmpR, *His4*, P*_HTX1_*-Cas9/HH-Yps7 (N20)-HDV | This study |
| pPIC3.5K-Mkc7-gRNA | pPIC3.5K, Zeocin, AmpR, *His4*, P*_HTX1_*-Cas9/HH-Mkc7 (N20)-HDV | This study |
| pPIC3.5K-Yps1-2-gRNA | pPIC3.5K, Zeocin, AmpR, *His4*, P*_HTX1_*-Cas9/HH-Yps1-2 (N20)-HDV | This study |
| pPIC3.5K-Yps1-5-gRNA | pPIC3.5K, Zeocin, AmpR, *His4*, P*_HTX1_*-Cas9/HH-Yps1-5 (N20)-HDV | This study |
| pPIC3.5K-Prc1-gRNA | pPIC3.5K, Zeocin, AmpR, *His4*, P*_HTX1_*-Cas9/HH-Prc1 (N20)-HDV | This study |
| pPIC3.5K-Pff1-gRNA | pPIC3.5K, Zeocin, AmpR, *His4*, P*_HTX1_*-Cas9/HH-Pff1 (N20)-HDV | This study |
| pPIC3.5K-Pep4-gRNA | pPIC3.5K, Zeocin, AmpR, *His4*, P*_HTX1_*-Cas9/HH-Pep4 (N20)-HDV | This study |
| pUC19-Donor-Yps1-*rhColIII* | pUC19, AmpR, Up Homologous arm (Yps1)-P*_AOX1_-rhColIII-*T*_AOX1_-* Up Homologous arm (Yps1) | This study |
| pET28a-ID (*KpSub2*) | pET28a, KanR, truncated *KpSub2* | This study |

**Table S4. The primers used in the article**

| **Primer name** | **Sequence 5‘-3’** |
| --- | --- |
| qPCR-*rhColI*-F | AACCCGGTGAACAAGGTGTT |
| qPCR-*rhColI*-R | GAGCACCATTAGCACCCCTT |
| qPCR-*rhColIII*-F | GAATCTGGTCCTGCCGGTAG |
| qPCR-*rhColIII*-R | AATACCAGCAGCGCCTCTTT |
| qPCR-*GAPDH*-F | CAAGTACGACTCTACCCACA |
| qPCR-*GAPDH*-R | GATGTTGACAGGGTCTCTCTCTTGG |
| 9K-GJ-F | TAAGGGAGAGCGTCGAGTATCTATGA |
| 9K-GJ-R | CATAGATACTCGACGCTCTCCCTTA |
| rhColI-F | GAGGCTGAAGCTGGTCCATCTGGTCCAAGAGGTT |
| rhColI-R | TGTCTAAGGCGAATTAGTTCAAACCATCTTTTCCAGGAG |
| rhColIII-F | GAGGCTGAAGCTGGTGCTAGAGGTA |
| rhColIII-R | TGTCTAAGGCGAATTATGGAGCACCTGGAGG |
| His4-YZ-F | TGTCTTCCCCAATCACTTGAGTACG |
| rhColI-YZ-R | CCAGCAGCACCTCTACTACCG |
| rhColIII-YZ-R | ATCACCCTTAGGACCTGGTTCAC |
| Ori-gRNA-F | TACCTGTCCGCCTTTCTCCC |
| Ori-gRNA-R | GGGAGAAAGGCGGACAGGTA |
| Prb1-gRNA-F | AAACAAATCAAACATCGACTGATGAGTCCGTGAGGACGAAACGAGTAAGCTCGTCTCGATGTCAGATGTTCTGAAGTTTTAGAGCTAGAAATAG |
| Prb1-gRNA-R | TCGTCCTCACGGACTCATCAGTCGATGTTTGATTTGTTTAGGTAACTTGAACT |
| *Kp*Sub2-gRNA-F | AAACAAATCAAACTGGAACTGATGAGTCCGTGAGGACGAAACGAGTAAGCTCGTCTTCCAGGGTAGAGCATCTTGGTTTTAGAGCTAGAAATAG |
| *Kp*Sub2-gRNA-R | TCGTCCTCACGGACTCATCAGTTCCAGTTTGATTTGTTTAGGTAACTTGAACT |
| Yps1-gRNA-F | AAACAAATCAAACTTGATCTGATGAGTCCGTGAGGACGAAACGAGTAAGCTCGTCATCAAGATCGTAAACCACGTGTTTTAGAGCTAGAAATAG |
| Yps1-gRNA-R | TCGTCCTCACGGACTCATCAGATCAAGTTTGATTTGTTTAGGTAACTTGAACT |
| Yps2-gRNA-F | AAACAAATCAAAATTAACCTGATGAGTCCGTGAGGACGAAACGAGTAAGCTCGTCGTTAATGTGAGACACCGACGGTTTTAGAGCTAGAAATAG |
| Yps2-gRNA-R | TCGTCCTCACGGACTCATCAGGTTAATTTTGATTTGTTTAGGTAACTTGAACT |
| Yps7-gRNA-F | AAACAAATCAAAGCGTTGCTGATGAGTCCGTGAGGACGAAACGAGTAAGCTCGTCCAACGCCAAAGACCCCACAGGTTTTAGAGCTAGAAATAG |
| Yps7-gRNA-R | TCGTCCTCACGGACTCATCAGCAACGCTTTGATTTGTTTAGGTAACTTGAACT |
| Mkc7-gRNA-F | AAACAAATCAAATCCCTCCTGATGAGTCCGTGAGGACGAAACGAGTAAGCTCGTCGAGGGATTCTTTCAACCAAGGTTTTAGAGCTAGAAATAG |
| Mkc7-gRNA-R | TCGTCCTCACGGACTCATCAGGAGGGATTTGATTTGTTTAGGTAACTTGAACT |
| Yps1-2-gRNA-F | AAACAAATCAAACCCTCACTGATGAGTCCGTGAGGACGAAACGAGTAAGCTCGTCTGAGGGCTGTCAATACCAAGGTTTTAGAGCTAGAAATAG |
| Yps1-2-gRNA-R | TCGTCCTCACGGACTCATCAGTGAGGGTTTGATTTGTTTAGGTAACTTGAACT |
| Yps1-5-gRNA-F | AAACAAATCAAACTGAACCTGATGAGTCCGTGAGGACGAAACGAGTAAGCTCGTCGTTCAGTTCTCCTTTGCAAGGTTTTAGAGCTAGAAATAG |
| Yps1-5-gRNA-R | TCGTCCTCACGGACTCATCAGGTTCAGTTTGATTTGTTTAGGTAACTTGAACT |
| Prc1-gRNA-F | AAACAAATCAAAGAAATTCTGATGAGTCCGTGAGGACGAAACGAGTAAGCTCGTCAATTTCTTGTCCAACAGCGAGTTTTAGAGCTAGAAATAG |
| Prc1-gRNA-R | TCGTCCTCACGGACTCATCAGAATTTCTTTGATTTGTTTAGGTAACTTGAACT |
| Pff1-gRNA-F | AAACAAATCAAACAACGACTGATGAGTCCGTGAGGACGAAACGAGTAAGCTCGTCTCGTTGGCTTTGGAGTCGAAGTTTTAGAGCTAGAAATAG |
| Pff1-gRNA-R | TCGTCCTCACGGACTCATCAGTCGTTGTTTGATTTGTTTAGGTAACTTGAACT |
| Pep4-gRNA-F | AAACAAATCAAACCCAGTCTGATGAGTCCGTGAGGACGAAACGAGTAAGCTCGTCACTGGGCAAAGCAATCAATGGTTTTAGAGCTAGAAATAG |
| Pep4-gRNA-R | TCGTCCTCACGGACTCATCAGACTGGGTTTGATTTGTTTAGGTAACTTGAACT |

In the construction of the gRNA plasmid, the N20 sequence corresponding to the target gene is shown by underlining.

**Table S5.** The amino acid sequences of collagens used in this study

| **Collagen variant** | **Amino acid sequence** |
| --- | --- |
| rhColI | GPSGPRGLPGPPGAPGPQGFQGPPGEPGEPGASGPMGPRGPPGPPGKNGDDGEAGKPGRPGERGPPGPQGARGLPGMKGHRGFSGLDGAKGDAGPAGPKGEPGSPGENGAPGQMGPRGLPGERGRPGAPGPAGARGNDGATGPPGPTGPAGPPGPSGPQGPGGPPGPKGNSGEPGAPGSKGDTGAKGEPGPVGVQGPPGPAGEEGKRGARGEPGPTGLPGPPGERGGPGSRGAAGEPGKAGERGVPGPAGKDGEAGAQGPPGPAGPAGERGEQGPAGSPGFQGPAGPPGEAGKPGEQGVPGAPGPSGARGERGFPGERGVQGPPGPAGPRGANGAPGNDGAKGDAGPKGDRGDAGPKGADGSPGKDGVRGRVGPPGPSGNAGPPGPPGPAGKEGGKGPRGETGPAGRPGEVGPPGPPGPAGEKGSPGADGPAGAPGTPGPQGPQGPRGDKGETGEQGDRGIKGHRGFSGLQGPPGPPGSPGEQGPSGASGPAGPRGPPGSAGAPGKDGLN |
| rhColIII | GARGNDGARGSDGQPGPPGPPGAKGEVGPAGSPGSNGAPGQRGEPGPQGHAGAQGPPGPPGINGSPGGKGEMGAAGERGAPGFRGPAGPNGIPGEKGPAGERGAPGPAGPRGAAGEPGRDGVPGGPGMRGMPGSPGGPGSDGKPGPPGSQGESGRPGPPGPSGPRGQPGPKGNDGAPGKNGERGGPGGPGPQGPPGKNGETGPQGPPGPTGPGGDKGDTGPPGPQGTGGPPGENGKPGEPGPKGDAGAPGGKGDAGAPGERGPPGPEGGKGAAGPPGLQRMPGERGGLGSPGPKGDKGEPGGPGADGVPGKDGPRGPTGPIGPPGPAGQPGDKGEGGAPGEPGRDGNPGSDGLPGRDGSPGGKGDRGENGSPGAPGAPGHPGPPGPVGPAGKSGDRGESGPAGSRGAPGPQGPRGDKGETGERGAAGIKGHRGFPGNPGAPGSPGPAGQQGPPGKDGTSGHPGPIGPPGPRGNRGERGSEGSPGHPGQPGPPGPPGAP |
| rhColIV | gtpgekgvpgipgpqgspglpgdkgakgekgtpgekgvpgipgpqgspglpgdkgakgekgtpgekgvpgipgpqgspglpgdkgakgekgtpgekgvpgipgpqgspglpgdkgakgekgtpgekgvpgipgpqgspglpgdkgakgekgtpgekgvpgipgpqgspglpgdkgakgekgtpgekgvpgipgpqgspglpgdkgakgekgtpgekgvpgipgpqgspglpgdkgakgekgtpgekgvpgipgpqgspglpgdkgakgekgtpgekgvpgipgpqgspglpgdkgakgek |
| rhColVII | GFDGQPGPKGDQGEKGERGTPGIGGFDGQPGPKGDQGEKGERGTPGIGGFDGQPGPKGDQGEKGERGTPGIGGFDGQPGPKGDQGEKGERGTPGIGGFDGQPGPKGDQGEKGERGTPGIGGFDGQPGPKGDQGEKGERGTPGIGGFDGQPGPKGDQGEKGERGTPGIGGFDGQPGPKGDQGEKGERGTPGIGGFDGQPGPKGDQGEKGERGTPGIGGFDGQPGPKGDQGEKGERGTPGIG |


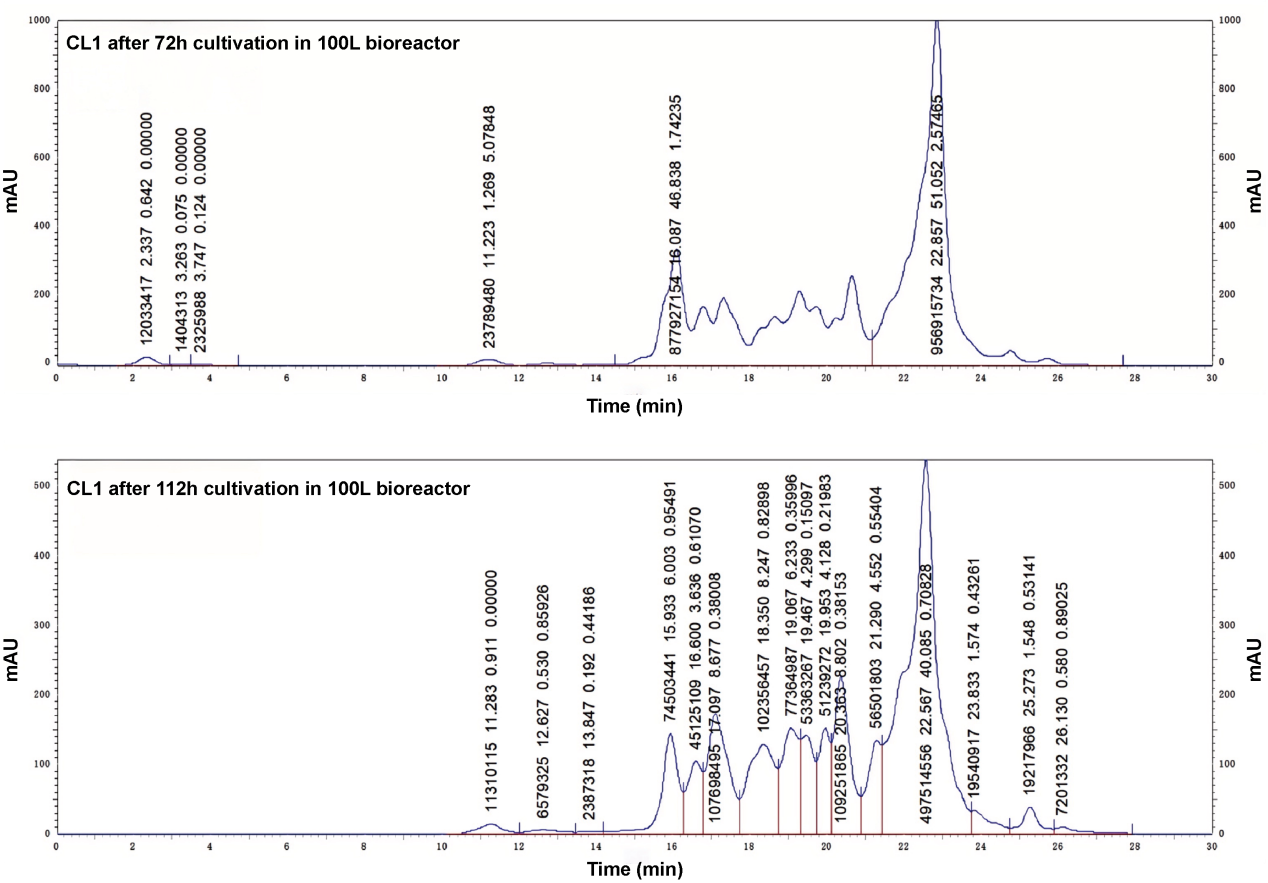


**Fig. S1 HPLC analysis of rhColI degradation in a 100 L bioreactor. a.** Representative chromatography result of strain CL1 after 72 h of fermentation, showing the intact rhColI peak (retention time ~16 min). b. Representative chromatography result after 112 h fermentation. The progressive degradation of the full-length protein is evident.


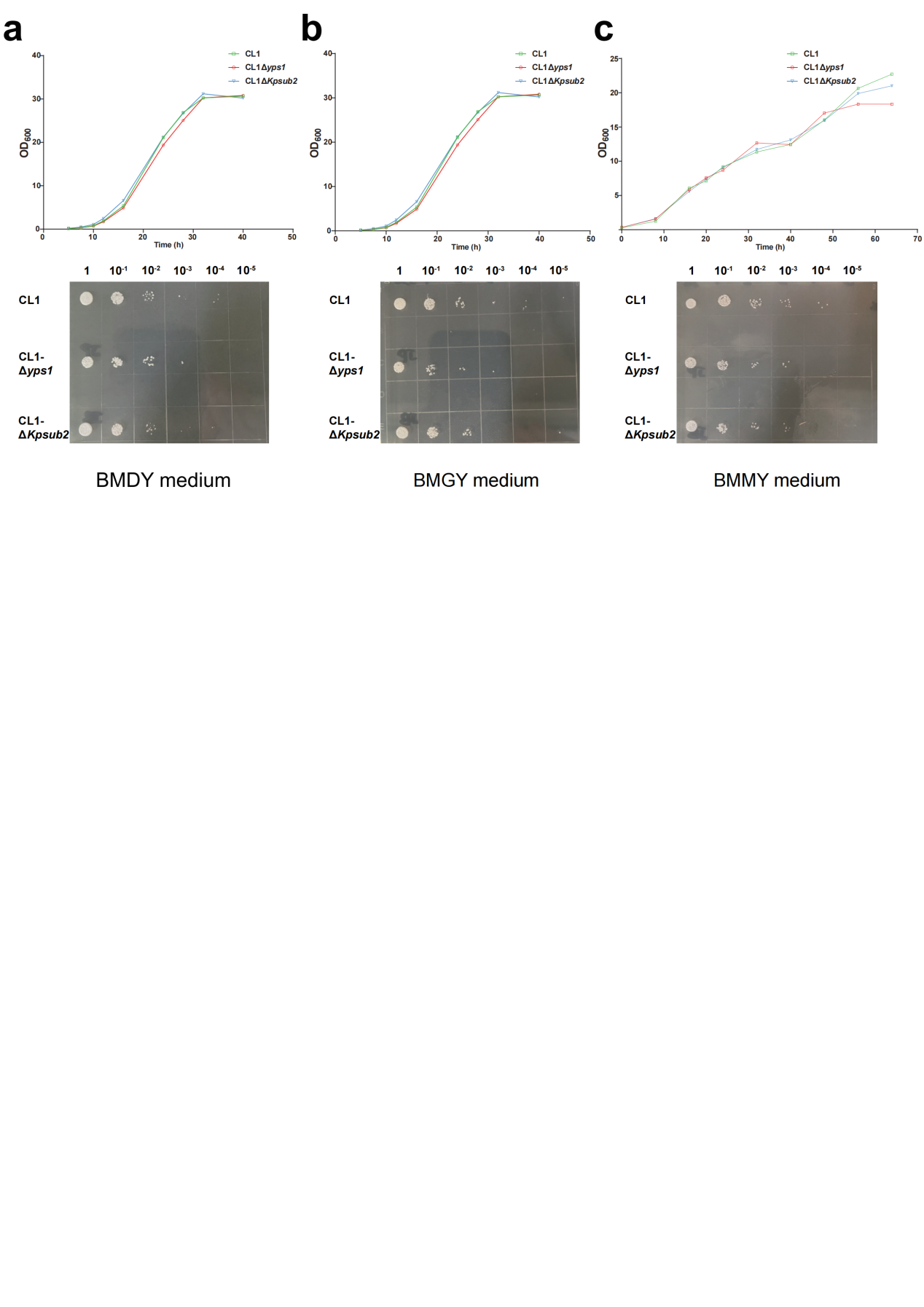


**Fig. S2** **Growth profiles of the parent strain CL1 and its derived *KpSub2*- and *Yps1*-deficient mutants.** **a–c.** Growth performance in BMDY (**a**), BMGY (**b**), and BMMY (**c**) media, respectively.


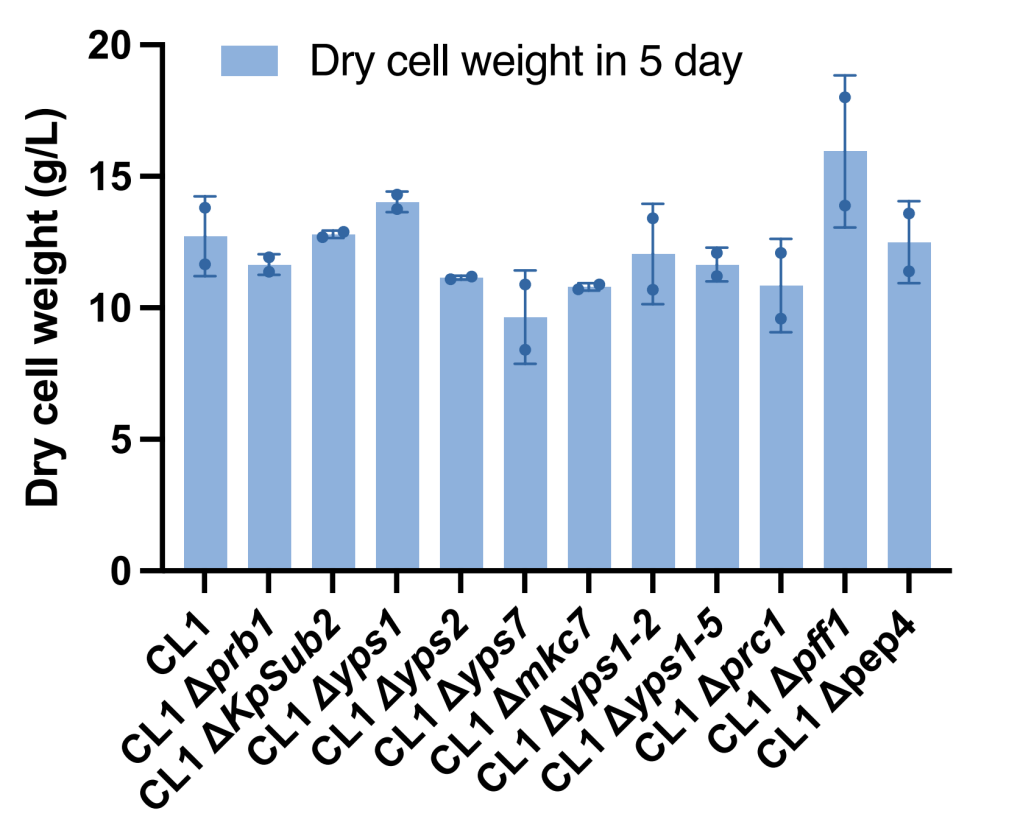


**Fig. S3 Biomass accumulation in 5 day for different protease-deficient strains in BMMY medium.**
